# Two New Secondary Metabolites from the Endophytic Fungus *Endomelanconiopsis endophytica*

**DOI:** 10.3390/molecules21070943

**Published:** 2016-07-20

**Authors:** Zhang-Hua Sun, Hao-Hua Li, Fa-Liang Liang, Yu-Chan Chen, Hong-Xin Liu, Sai-Ni Li, Guo-Hui Tan, Wei-Min Zhang

**Affiliations:** State Key Laboratory of Applied Microbiology Southern China, Guangdong Provincial Key Laboratory of Microbial Culture Collection and Application, Guangdong Open Laboratory of Applied Microbiology, Guangdong Institute of Microbiology, Guangzhou 510070, China; sysuszh@126.com (Z.-H.S.); hhli100@126.com (H.-H.L.); 15627860105@163.com (F.-L.L.); yuchan2006@126.com (Y.-C.C.); hxinliu1225@163.com (H.-X.L.); maibao66@126.com (S.-N.L.); 13430272007@163.com (G.-H.T.)

**Keywords:** endophytic fungus, *Endomelanconiopsis endophytica*, ketals, *Ficus hirta*

## Abstract

Two new secondary metabolites, endomeketals A–B (**1**–**2**), a new natural product (**3**), and a known compound (**4**) were isolated from the ethyl acetate extract of the endophytic fungus *Endomelanconiopsis endophytica* A326 derived from *Ficus hirta*. Their structures were determined on the basis of extensive spectroscopic analysis. All compounds were evaluated for their cytotoxic activities against SF-268, MCF-7, NCI-H460, and HepG-2 tumor cell lines. However, no compound showed cytotoxic activity against these human tumor cell lines.

## 1. Introduction

Endophytes are microorganisms that reside in the tissues of living plants asymptomatically, and the mutualistic interaction that occurs between the two may have benefits to each other. These organisms represent a huge and largely untapped resource of natural products with chemical structures that have been optimized by evolution for biological and ecological relevance [1]. Over the past 20 years, a surprisingly high number of metabolites have been described from endophytes [2,3,4,5,6,7,8]. Several secondary metabolites, including alkaloids [9], sesquiterpenoids [10], and azaphilones [11], exhibit a variety of biological activities. *Ficus hirta* Vahl. (Moraceae), endemic to Guangdong province of China, is known as “Wu Zhi Mao Tao” in traditional Chinese medicine for the treatment of cough, asthma, and inflammatory diseases [12]. As part of an ongoing program aimed at exploring bioactive secondary metabolites from endophytic fungi [13,14,15], we undertook a detailed chemical analysis on the ethyl acetate (EtOAc) extract of *Endomelanconiopsis endophytica* A326 derived from the medicinal plant *Ficus hirta*, which led to the isolation of two new secondary metabolites (**1**–**2**) and two known compounds (**3**–**4**). All compounds were evaluated for their cytotoxic activities against SF-268, MCF-7, NCI-H460, and HepG-2 tumor cell lines. Herein, details of the isolation and structural elucidation of these compounds are described.

## 2. Results and Discussion

**Figure 1:**
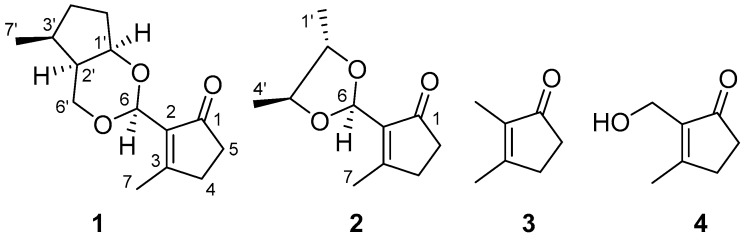
The chemical structures of compounds **1**–**4**.

The liquid culture of the fungus *E. endophytica* was centrifuged to separate broth and mycelia. The broth was exhaustively extracted with ethyl acetate (EtOAc), and the concentrated EtOAc extract was further purified by various chromatographic methods to yield four compounds (**1**–**4**) (Figure 1). Two new compounds, endomeketals A–B (**1**–**2**), were identified by spectroscopic analyses and physicochemical properties, while a new natural product and a known compound were identified as 2,3-dimethylcyclopent-2-en-1-one (**3**) [16,17] and 2-hydroxymethyl-3-methylcyclopent-2-enone (**4**) [18] by comparison of their spectroscopic data with those in the literature.

### 2.1. Identification of New Compounds

Compound **1** was assigned a molecular formula of C_14_H_20_O_3_ on the basis of ^13^C nuclear magnetic resonance (NMR) data and high resolution electrospray ionization mass spectroscopy (HRESIMS) ion at *m*/*z* 259.1308 [M + Na]^+^ (calcd 259.1310). The ^1^H-NMR spectrum (Table 1) showed one methyl doublet [δ_H_ 1.04 (d, *J* = 6.7 Hz, H_3_-7′)], one vinylic methyl [δ_H_ 2.31 (H_3_-7)], four protons bonded to carbons bearing heteroatoms (δ_H_ 5.36 (s, H-6), 4.25 (dd, *J* = 4.2, 4.1 Hz, H-1′), 4.07 (dd, *J* = 11.9, 3.1 Hz, H-6′a), and 4.03 (d, *J* = 11.9 Hz, H-6′b), and a series of aliphatic methylene multiplets (Figure S1). The ^13^C-NMR spectrum (Figure S2), in combination with heteronuclear single quantum coherence (HSQC, Figure S3) experiments, showed 14 carbon resonances attributable to one carbonyl (δ_C_ 206.8, C-1), two sp^2^ quaternary carbons (δ_C_ 177.8 (C-3) and 137.4 (C-2), four sp^3^ methines [δ_C_ 94.4 (C-6), 81.0 (C-1′), 47.2 (C-2′), and 32.5 (C-3′)], five methylenes [δ_C_ 66.3 (C-6′), 34.4 (C-5), 32.4 (C-4), 31.8 (C-4′), and 31.4 (C-5′)], and two methyls [δ_C_ 19.0 (C-7′) and 18.3 (C-7)]. As two of the five degrees of unsaturation were accounted for by a double bond and a carbonyl group, the remaining three degrees of unsaturation required that **1** was tricyclic.

**Figure 2:**
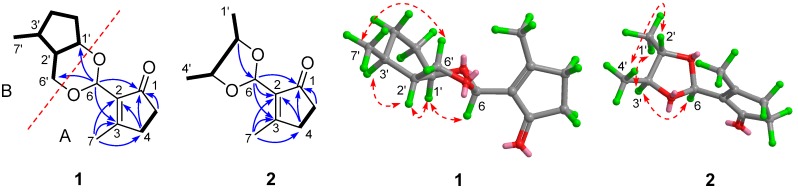
Key ^1^H-^1^H-COSY (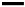), HMBC (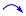), and NOE (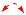) correlations of compounds **1**–**2**.

Detailed 2D-NMR studies (HSQC, ^1^H-^1^H-COSY, and HMBC experiments) afforded the gross structures of two sub-units (A and B) as depicted in Figure 2. ^1^H-^1^H-COSY correlation between H-4 and H-5 and HMBC correlations from H_3_-7 to C-2/C-3/C-4, from H-5 to C-1, and from H-6 to C-1/C-2/C-3, revealed unit A was highly similar to 2-hydroxymethyl-3-methylcyclopent-2-enone (**4**) (Figure 2), which was further confirmed by comparison of their 1D-NMR data. As for unit B, the structural fragments (C-1′→C-7′) were established by the ^1^H-^1^H-COSY correlations of C-1′/C-2′/C-3′/C-4′/C-5′/C-1′, and C-2′/C-6′ and C-3′/C-7′. The connectivities of two sub-units were mainly achieved by HMBC interactions from H-6 to C-1′ and C-6′. The relative configuration of **1** was determined by a NOESY experiment. NOESY correlations of H-6/H-1′/H-2′/H-3′ and Me-7′/H-6′ indicated that H-6, H-1′, H-2′, and H-3′ were cofacial and designated as α-oriented (Figure S6). Thus, compound **1** was determined as depicted and given the trivial name endomeketal A.

HRESIMS analysis of **2** revealed an adduct ion [M + Na]^+^ consistent with a molecular formula (C_11_H_16_O_3_) requiring four double bond equivalents (Figure S14). The 1D-NMR data of **2** were similar to those of **1** except for the absence of signals for the saturated pentacyclic unit and the presence of the signals for two methyls and two sp^3^ methines bearing heteroatoms (Table 1, Figures S8 and S9). The gross structure of **2** was established by analyses of its 2D-NMR data (Figures S10–S13). COSY correlations revealed a ^1^H spin system from C-1′ to C-4′, so that the sub-structure B in **1** was substituted in **2** by a butane-2,3-diyl residue. The carbon chemical shifts assigned to unit A in **2** were nearly identical to the corresponding carbon chemical shifts assigned to this fragment in **1**. The HMBC interactions from H-6 to C-1, C-2, and C-3, and from H-2′ to C-6 connected unit B to unit A. As three of the four degrees of unsaturation were accounted for by the unsaturated pentacyclic unit, the remaining double bond equivalent required that unit B and unit A in **2** were connected via a 1,3-dioxolane. The relative configuration of **2** was determined by a NOESY experiment. NOESY correlations of H-6/H-3′ and H-3′/H_3_-1′ indicated that H_3_-1′, H-3′, and H-6 were cofacial and designated as α-oriented, while correlation of H-2′/H_3_-4′ assigned H-2′ and H_3_-4′ as β. Thus, compound **2** was assigned as depicted and named endomeketal B.

By comparison of their spectroscopic data (Figures S15–S18) with those reported [16,17,18], the structures of compounds **3** and **4** were assigned as 2,3-dimethylcyclopent-2-en-1-one (**3**) and 2-hydroxymethyl-3-methylcyclopent-2-enone (**4**), respectively. Although compound **3** was previously reported as a synthetic compound, this is the first report of its isolation from a natural source.

### 2.2. Cytotoxicity Assay

The in vitro cytotoxicities of compounds **1**–**4** were evaluated against four tumor cell lines, including SF-268 (human glioblastoma cell line, ATCC HTB-14), MCF-7 (human breast adenocarcinoma cell line, ATCC HTB-22), NCI-H460 (human non-small cell lung cancer cell line, ATCC HTB-177), and HepG-2 (human hepatocellular carcinoma cell line, ATCC HB-8065). However, no compound showed obvious cytotoxic activity against these human tumor cell lines at the concentration of 100 µM.

## 3. Materials and Methods

### 3.1. General Experimental Procedures

Optical rotation was measured on an Anton Paar MCP-500 spectropolarimeter (Anton Paar, Vienna, Austria). The IR spectrum was recorded on an IRAffinity-1 spectrophotometer in cm^−1^ (Shimadzu Corporation, Kyoto, Japan). UV spectra were measured on a SHIMADZU UV-2600 UV-VIS spectrophotometer (Shimadzu Corporation). 1D- and 2D-NMR spectra were recorded on a Bruker Avance-600 spectrometer with TMS as internal standard (Bruker BioSpin International, Geneva, Switzerland), δ in ppm, *J* in Hz. HRESIMS was measured on a Thermo MAT95XP high-resolution mass spectrometer. A Shimadzu LC-20 AT (Shimadzu Corporation) equipped with an SPD-M20A PDA detector (Shimadzu Corporation) was used for HPLC, a YMC-pack ODS-A column (250 m × 10 mm, 5 µm, 12 nm) was used for semi-preparative HPLC separation. Silica gel (200–300 mesh) was used for column chromatography, and precoated silica gel GF_254_ plates (Qingdao Marine Chemical Inc., Qingdao, China) were used for TLC spotting. All solvents were analytical grade (Guangzhou Chemical Reagents Company, Ltd., Guangzhou, China).

### 3.2. Fungal Material

The endophytic fungal strain A326 was isolated from the twigs of *Ficus hirta*, which was collected at Luofu Mountain Nature Reserve, Huizhou, Guangdong province of China, in October 2010. The isolated strain was identified as *Endomelanconiopsis endophytica* based on a morphological study, and sequence analysis of rDNA ITS (internal transcribed spacer), showing 100% similarity to the strain of *E. endophytica* (Accession No. EU687005). The strain is preserved at the Guangdong Provincial Key Laboratory of Microbial Culture Collection and Application, Guangdong Institute of Microbiology.

### 3.3. Extraction and Isolation

*E. endophytica* A326 was grown on potato-dextrose agar (PDA) medium at 28 °C for five days and then inoculated into five flasks (500 mL) containing potato-dextrose (PD) medium (250 mL). After five days of incubation at 28 °C on a rotary shaker at 120 r/min, a portion of the liquid culture was aseptically transferred into each of a total of 200 flasks (500 mL) containing potato-dextrose (PD) medium (250 mL). Following seven days of cultivation at 28 °C and 120 r/min on a rotary shaker, the culture (a total of 50 L) was filtered to give the filtrate and mycelia. The broth was exhaustively extracted with ethyl acetate (EtOAc) for four times, and then the EtOAc layers were combined and evaporated under reduced pressure at a temperature not exceeding 40 °C to yield a dark brown gum (11.7 g). The crude extract was subjected to silica gel column chromatography with a CH_2_Cl_2_/MeOH gradient (9:1→1:9) to afford six fractions (Fr. I–VI). Fr. II (1.1 g) was subjected to column chromatography on silica gel using *n*-hexane as the first eluent, and then EtOAc of increasing polarity, to give nine sub-fractions (Fr. II_a_−Fr. II_i_). Fr.II_d_ was further separated by RP-HPLC equipped with a semi-preparative column (CH_3_CN, 3 mL/min) to afford **2** (5 mg, *t*_R_ 7.6 min) and **3** (19 mg, *t*_R_ 9.1 min). Fr. II_e_ was chromatographed by RP-HPLC equipped with a semi-preparative column (MeOH/H_2_O, 70:30, 3 mL/min) to afford **4** (36 mg, *t*_R_ 7.2 min) and **1** (24 mg, *t*_R_ 13.0 min).

### 3.4. Spectroscopic Data

*Endomeketal A* (**1**): white, amorphous powder (MeOH); ${\left[\alpha \right]}_{D}^{25}$ +33.8 (*c* 0.5, MeOH); UV (MeOH) λ_max_ (log ε) 229 (3.83) nm; IR (KBr) ν_max_ = 1693, 1435, 1409, 1259, 1092, 1018 cm^−1^; HRESIMS *m*/*z* 259.1308 [M + Na]^+^ (calcd for C_14_H_20_O_3_Na, 259.1310, Figure S7); ^1^H- and ^13^C-NMR data, see Table 1.

*Endomeketal B* (**2**): white, amorphous powder (MeOH); ${\left[\alpha \right]}_{D}^{25}$ +23.0 (*c* 1.0, MeOH); UV (MeOH) λ_max_ (log ε) 222 (3.56) nm; IR (KBr) ν_max_ = 1691, 1381, 1257, 1087, 1022 cm^−1^; HRESIMS *m*/*z* 219.1002 [M + Na]^+^ (calcd for C_11_H_16_O_3_Na, 219.0997, Figure S14); ^1^H- and ^13^C-NMR data, see Table 1.

### 3.5. Cytotoxicity Assay

The cell growth inhibitory activities of compounds **1**–**4** against human tumor cell lines SF-268, MCF-7, NCI-H460, and HepG-2, were tested using the previously-published method [19].

## 4. Conclusions

Fungal endophytes live inside plant tissues for all, or part, of their life without causing apparent disease symptoms. Endophytic fungi have been found within host tissues at high density and diversity in all tropical and temperate ecosystems where endophytes have been sought [20]. *Endomelanconiopsis* is a new anamorph genus in the Botryosphaeriaceae, and usually lives inside plant tissues [21]. Previous investigation onto *E. endophytica* A326 in our research group led to the isolation of a series of xyloketals, which were the first chemical constituents of the genus *Endomelanconiopsis* [22]. As the fermentation products of the fungus *E. endophytica* displayed considerable structural diversity, continued investigation of this fungus has resulted in the isolation of two further new secondary metabolites, which were named as endomeketals A and B. The structures were determined by spectroscopic analysis. All of the isolates were evaluated for in vitro cytotoxicity against SF-268, MCF-7, NCI-H460, and HepG-2 cell lines. This study not only enriches the chemical diversity of the genus *Endomelanconiopsis*, but also implies that this endophytic fungus may be a potential source for producing a series of ketals.

## Figures and Tables

**Table 1:** ^1^H (500 MHz) and ^13^C (125 MHz)-NMR data of **1**−**2** in CDCl_3_ (*J* in Hz, δ in ppm).

Position	1		2	
	δ_H_	δ_C_	δ_H_	δ_C_
1		206.8		207.1
2		137.4		136.0
3		177.8		177.5
4	2.52 (1H, m) 2.41 (1H, m)	32.4	2.55 (2H, ddd, 5.6, 2.3, 1.1)	32.5
5	2.36 (1H, m)	34.4	2.37 (1H, m)	34.5
6	5.36 (1H, s)	94.4	5.80 (1H, s)	95.9
7	2.31 (3H, s)	18.3	2.25 (3H, s)	17.8
1′	4.25 (1H, m)	81.0	1.27 (3H, d, 6.1)	17.3
2′	1.18 (1H, ddd, 10.7, 4.5, 3.0)	47.2	3.82 (1H, dd, 7.8, 6.1)	78.6
3′	2.42 (1H, m)	32.5	3.70 (1H, dd, 7.8, 6.0)	80.4
4′	2.12 (1H, m) 1.25 (1H, m)	31.8	1.36 (3H, d, 6.0)	16.5
5′	1.70 (1H, ddd, 14.3, 9.1, 4.9) 1.87 (1H, m)	31.4		
6′	4.04 (1H, d, 11.8) 4.07 (1H, dd, 11.8, 3.0)	66.3		
7′	1.04 (3H, d, 6.7)	19.0		

## Supplementary Materials

Supplementary materials can be accessed at: http://www.mdpi.com/1420-3049/21/7/943/s1.
