# Supplementary material for: Two New Secondary Metabolites from the Endophytic Fungus Endomelanconiopsis endophytica
Source: Molecules. 2016 Jul 20;21(7):943. doi: 10.3390/molecules21070943 (PMC6274285; doi:10.3390/molecules21070943)
Supplement: Supplementary file 1 [file molecules-21-00943-s001.pdf]

Supplementary Materials: Two New Secondary Metabolites from the Endophytic Fungus *Endomelanconiopsis endophytica*

Zhang-Hua Sun, Hao-Hua Li, Fa-Liang Liang, Yu-chan Chen, Hong-Xin Liu, Sai-Ni Li, Guo-Hui Tan and Wei-Min Zhang

List of SupplementaryMaterials

Figure S1. ^{1}H-NMR spectrum of endomeketal A (**1**) in $CDCl_3$
Figure S2. ^{13}C-NMR spectrum of endomeketal A (**1**) in $CDCl_3$
Figure S3. HSQC spectrum of endomeketal A (**1**) in $CDCl_3$
Figure S4. ^{1}H-^{1}H-COSY spectrum of endomeketal A (**1**) in $CDCl_3$
Figure S5. HMBC spectrum of endomeketal A (**1**) in $CDCl_3$
Figure S6. NOESY spectrum of endomeketal A (**1**) in $CDCl_3$
Figure S7. HRESIMS spectrum of endomeketal A (**1**)
Figure S8. ^{1}H-NMR spectrum of endomeketal B (**2**) in $CDCl_3$
Figure S9. ^{13}C-NMR spectrum of endomeketal B (**2**) in $CDCl_3$
Figure S10. HSQC spectrum of endomeketal B (**2**) in $CDCl_3$
Figure S11. ^{1}H-^{1}H-COSY spectrum of endomeketal B (**2**) in $CDCl_3$
Figure S12. HMBC spectrum of endomeketal B (**2**) in $CDCl_3$
Figure S13. NOESY spectrum of endomeketal B (**2**) in $CDCl_3$
Figure S14. HRESIMS spectrum of endomeketal B (**2**)
Figure S15. ^{1}H-NMR spectrum of 2,3-dimethylcyclopent-2-en-1-one (**3**) in $CDCl_3$
Figure S16. ^{13}C-NMR spectrum of 2,3-dimethylcyclopent-2-en-1-one (**3**) in $CDCl_3$
Figure S17. ^{1}H-NMR spectrum of 2-hydroxymethyl-3-methylcyclopent-2-enone (**4**) in $CDCl_3$
Figure S18. ^{13}C-NMR spectrum of 2-hydroxymethyl-3-methylcyclopent-2-enone (**4**) in $CDCl_3$

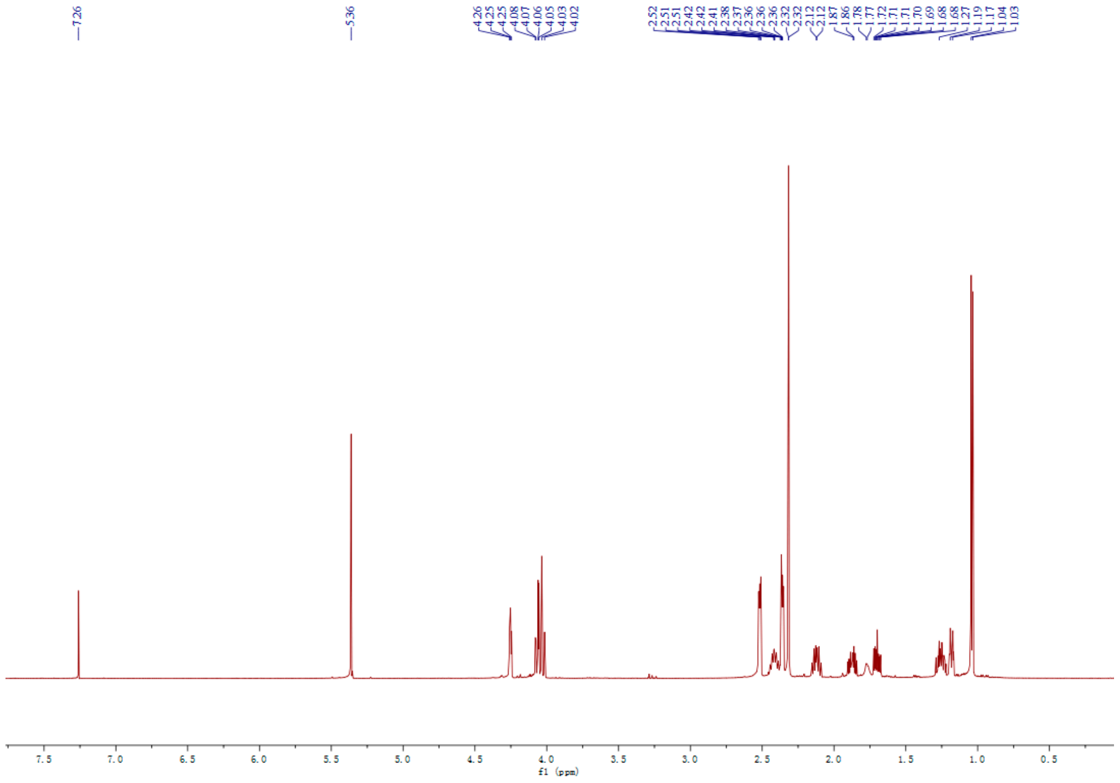

Figure S1. ^{1}H-NMR spectrum of endophyticketal A (**1**) in $CDCl_3$.

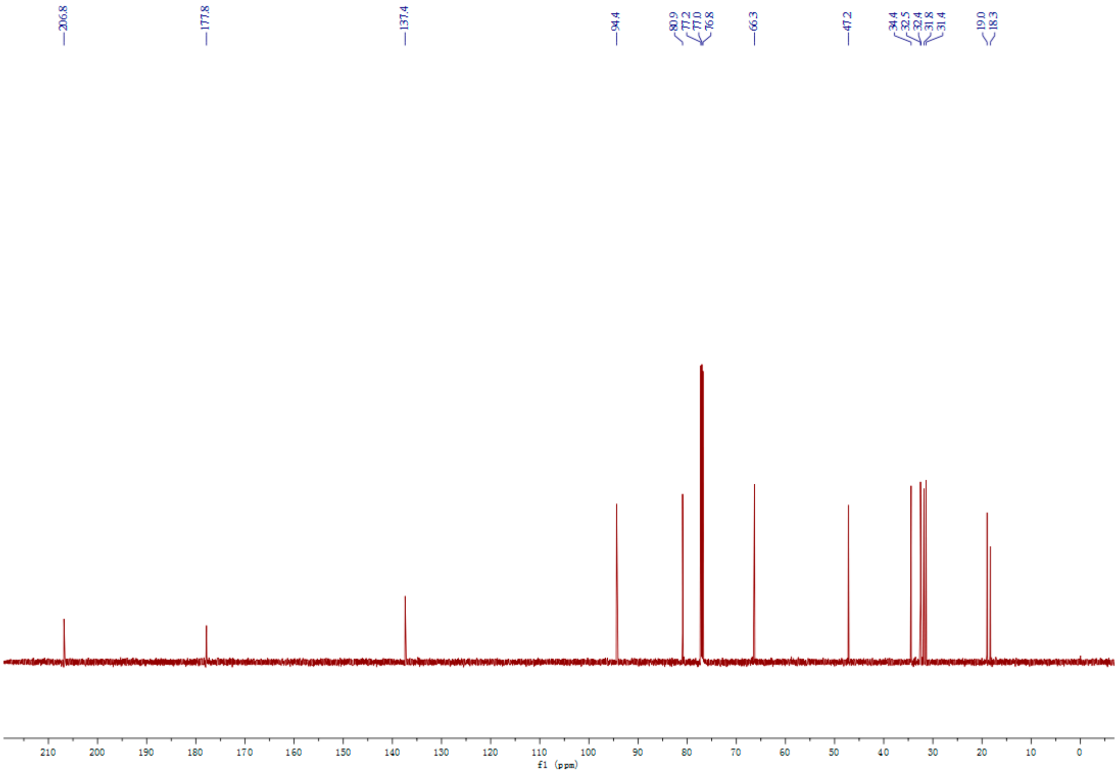

Figure S2. ^{13}C-NMR spectrum of endomeketal A (**1**) in $CDCl_3$.

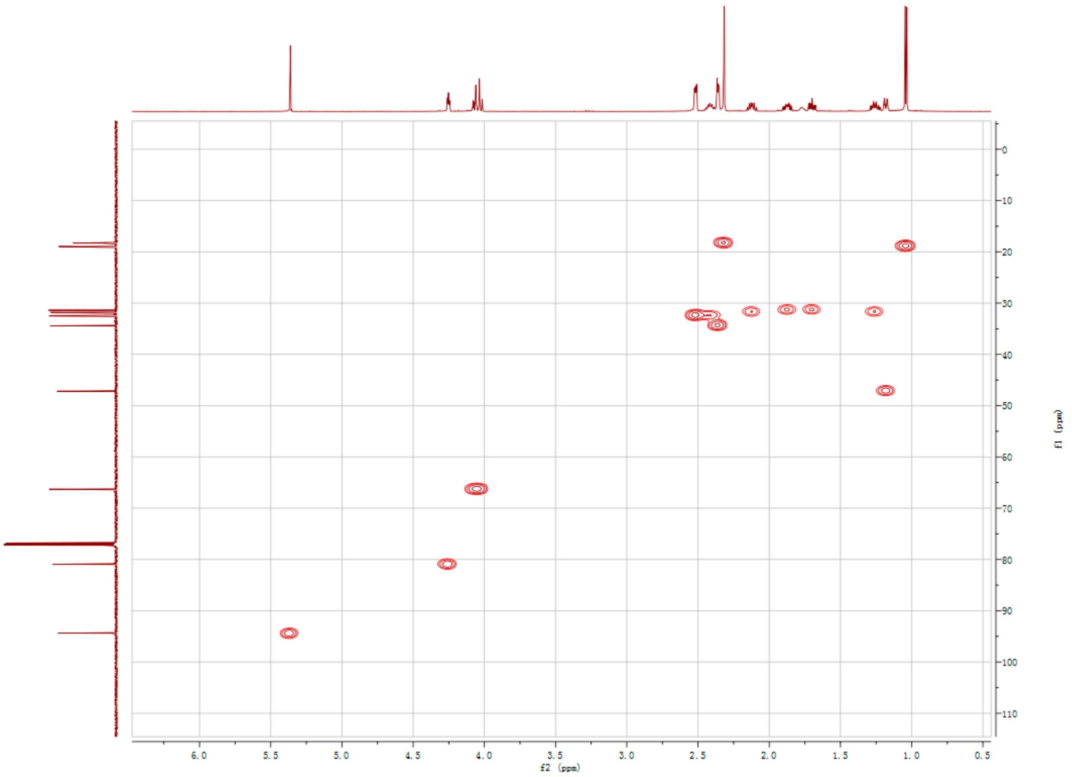

Figure S3. HSQC spectrum of endomeketal A (**1**) in $CDCl_3$.

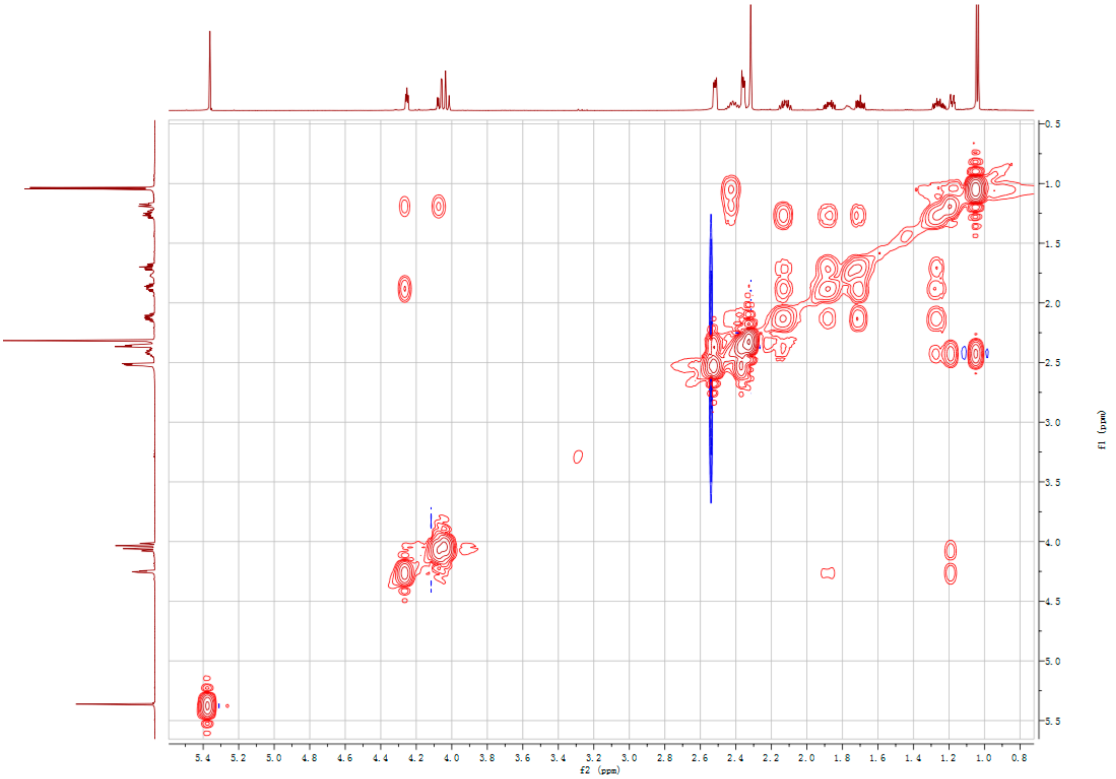

Figure S4. ${}^{1}H$-${}^{1}H$-COSY spectrum of endomeketal A (**1**) in $CDCl_3$.

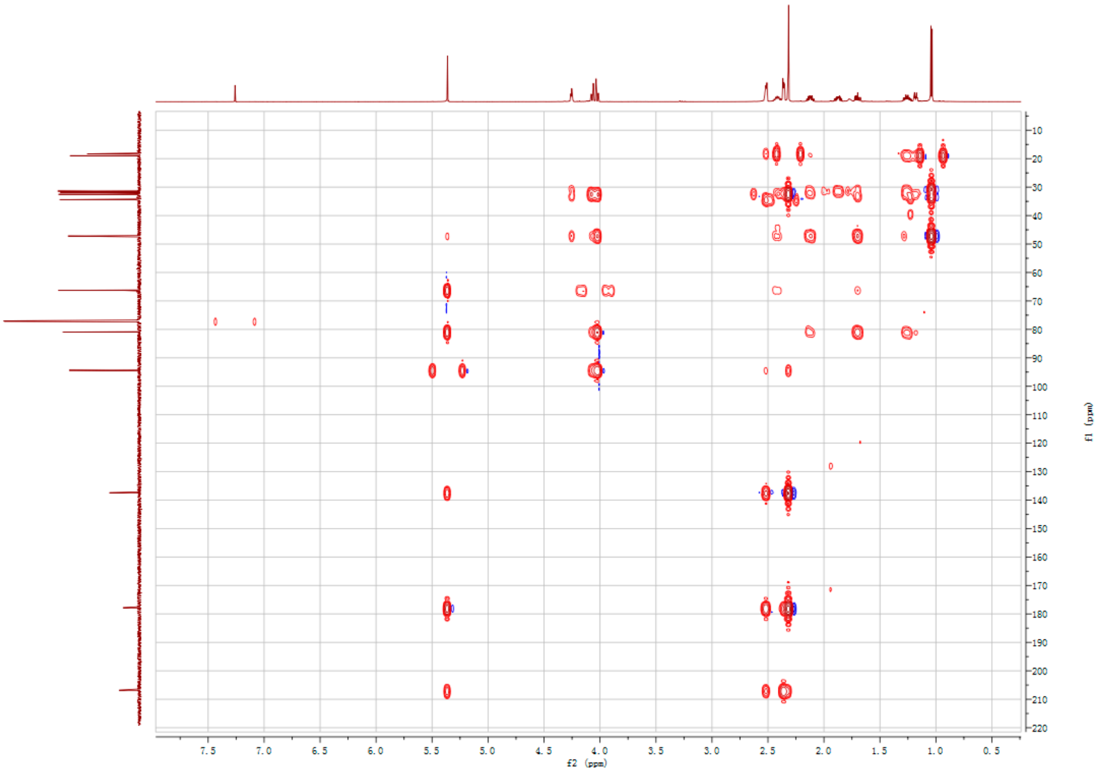

Figure S5. HMBC spectrum of endomeketal A (**1**) in $CDCl_3$.

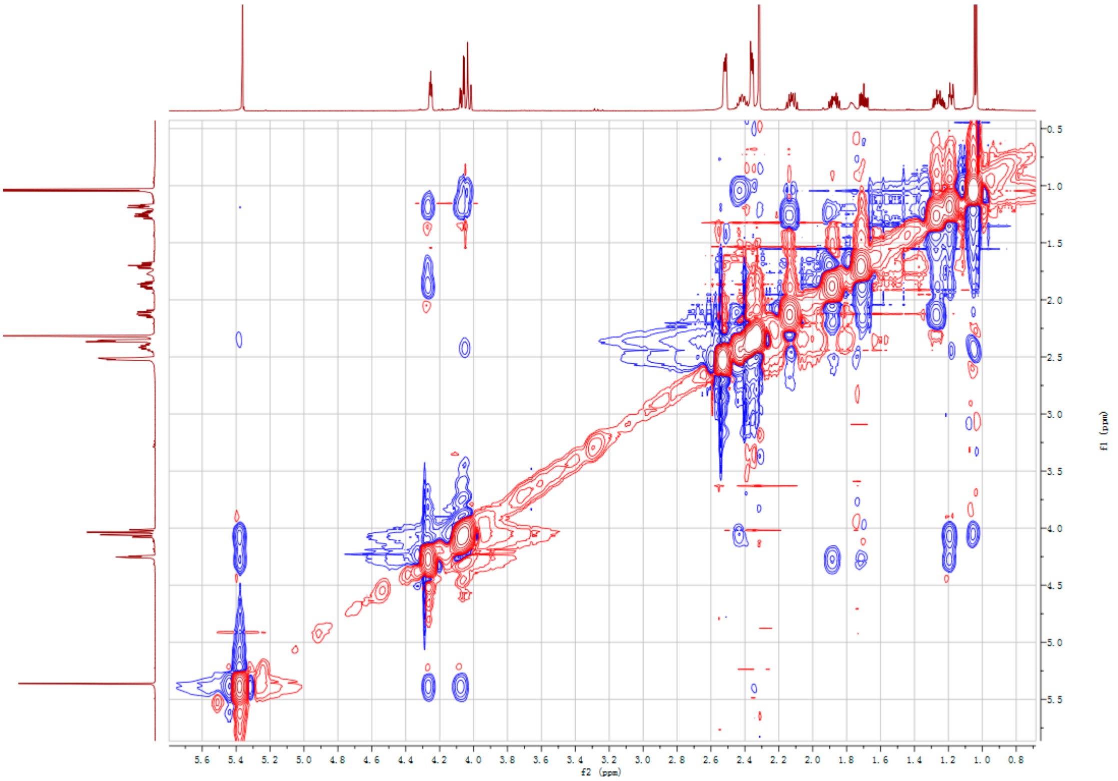

Figure S6. NOESY spectrum of endomeketal A (**1**) in $CDCl_3$.

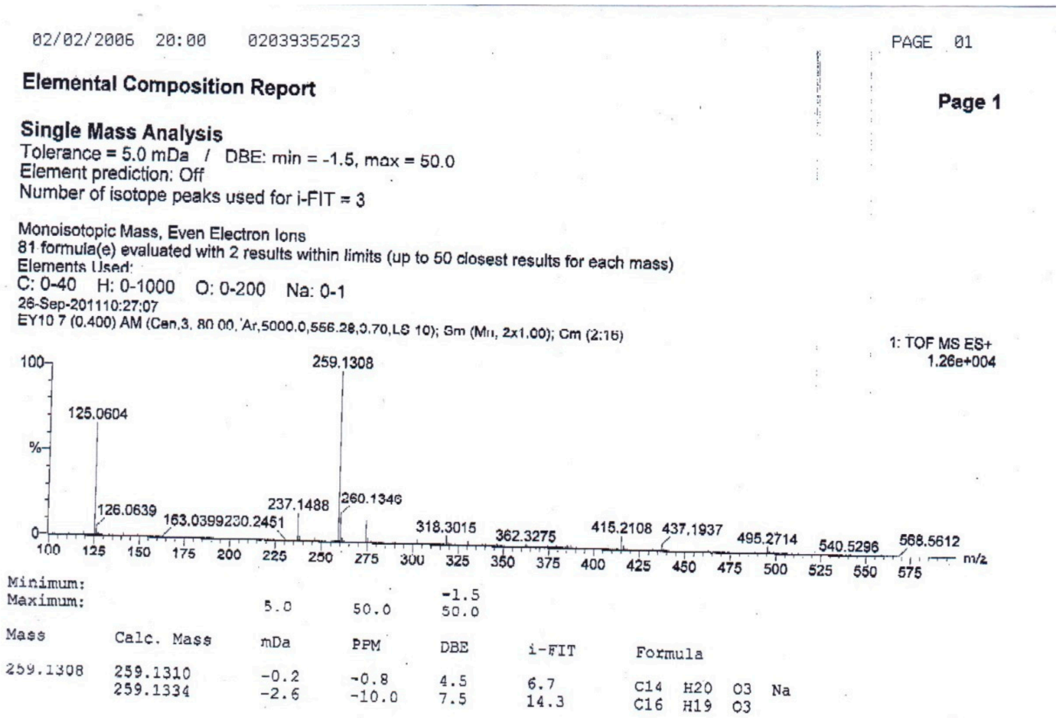

02/02/2006 20:00 02039352523 PAGE 01

Elemental Composition Report **Page 1**

Single Mass Analysis
Tolerance = 5.0 mDa / DBE: min = -1.5, max = 50.0
Element prediction: Off
Number of isotope peaks used for i-FIT = 3

Monoisotopic Mass, Even Electron Ions
81 formula(e) evaluated with 2 results within limits (up to 50 closest results for each mass)
Elements Used:
C: 0-40 H: 0-1000 O: 0-200 Na: 0-1
26-Sep-201110:27:07
EY10 7 (0.400) AM (Cen,3, 80.00, Ar,5000.0,556.28,0.70,LS 10); Sm (Mn, 2x1.00); Cm (2:16)

Minimum:				-1.5		
Maximum:		5.0	50.0	50.0		
Mass	Calc. Mass	mDa	PPM	DBE	i-FIT	Formula
259.1308	259.1310	-0.2	-0.8	4.5	6.7	C14 H20 O3 Na
	259.1334	-2.6	-10.0	7.5	14.3	C16 H19 O3

Figure S7. HRESIMS spectrum of endomeketal A (**1**).

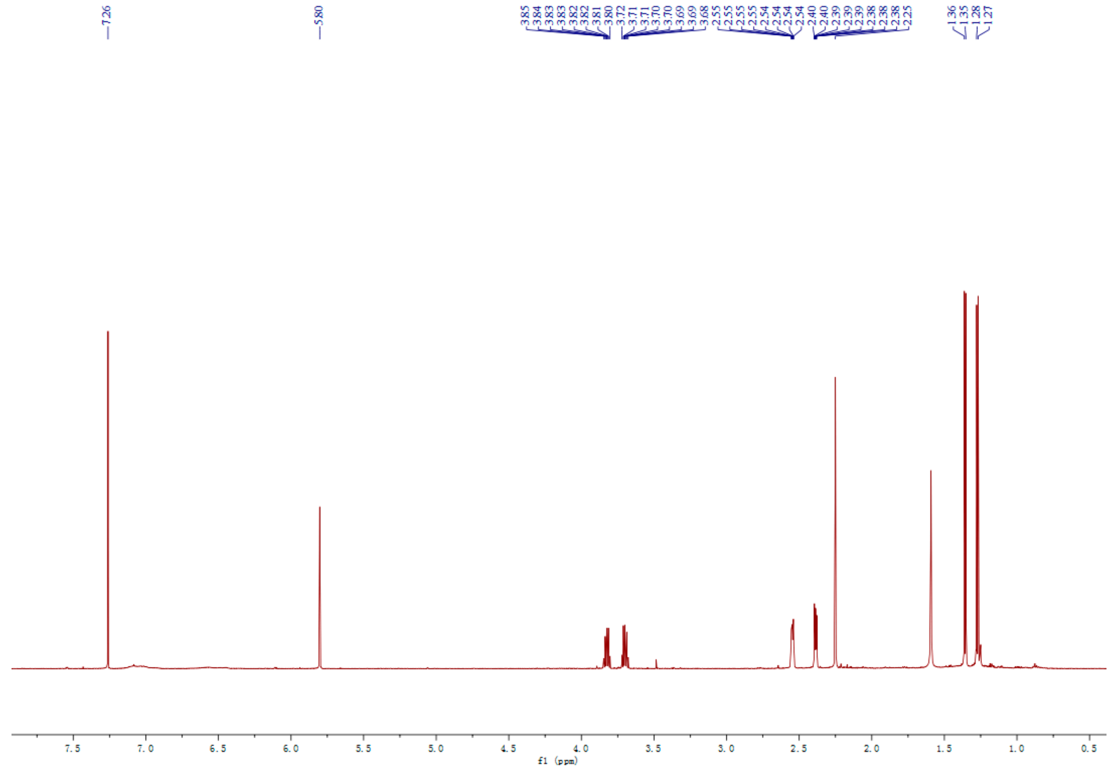

Figure S8. ^{1}H-NMR spectrum of endomeketal B (**2**) in $CDCl_3$.

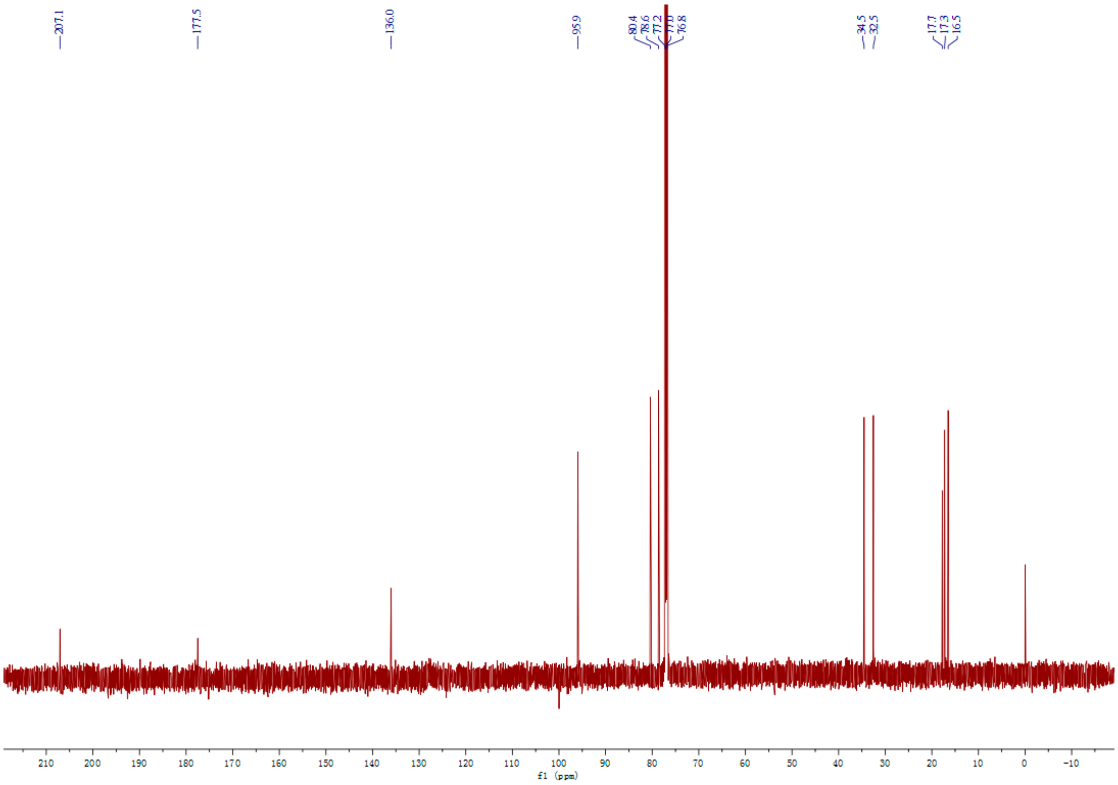

Figure S9. ^{13}C-NMR spectrum of endomeketal B (**2**) in $CDCl_3$.

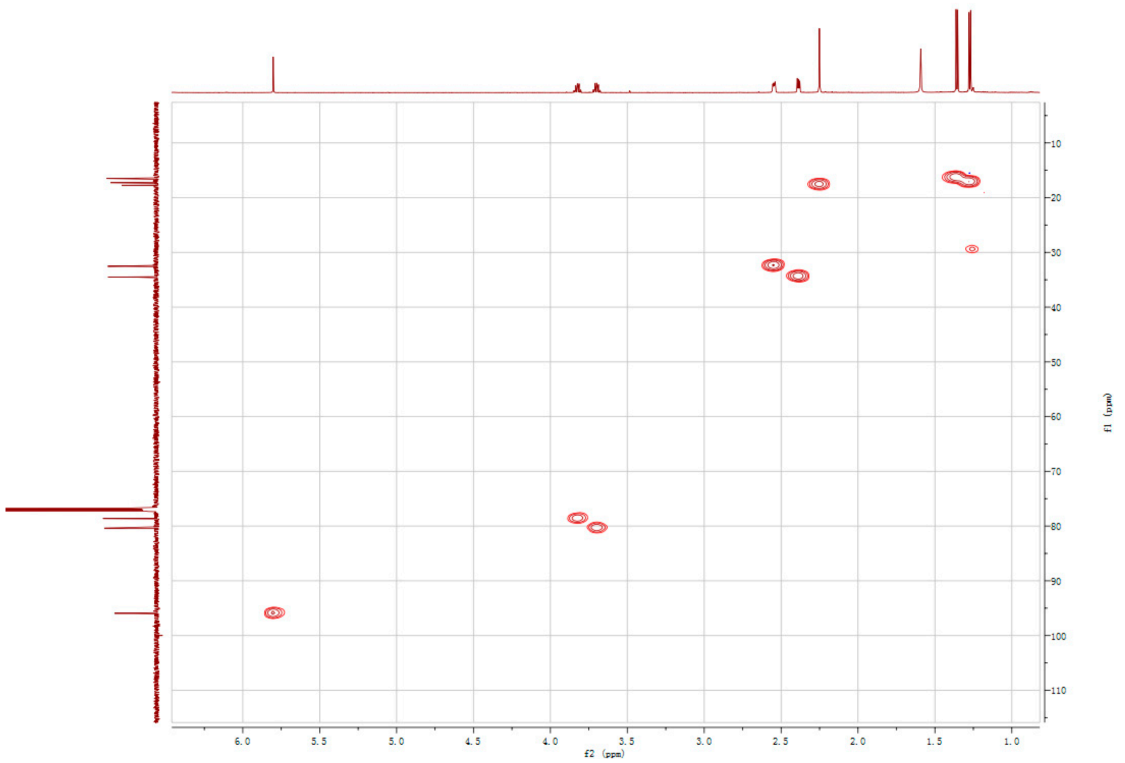

Figure S10. HSQC spectrum of endomeketal B (**2**) in $CDCl_3$.

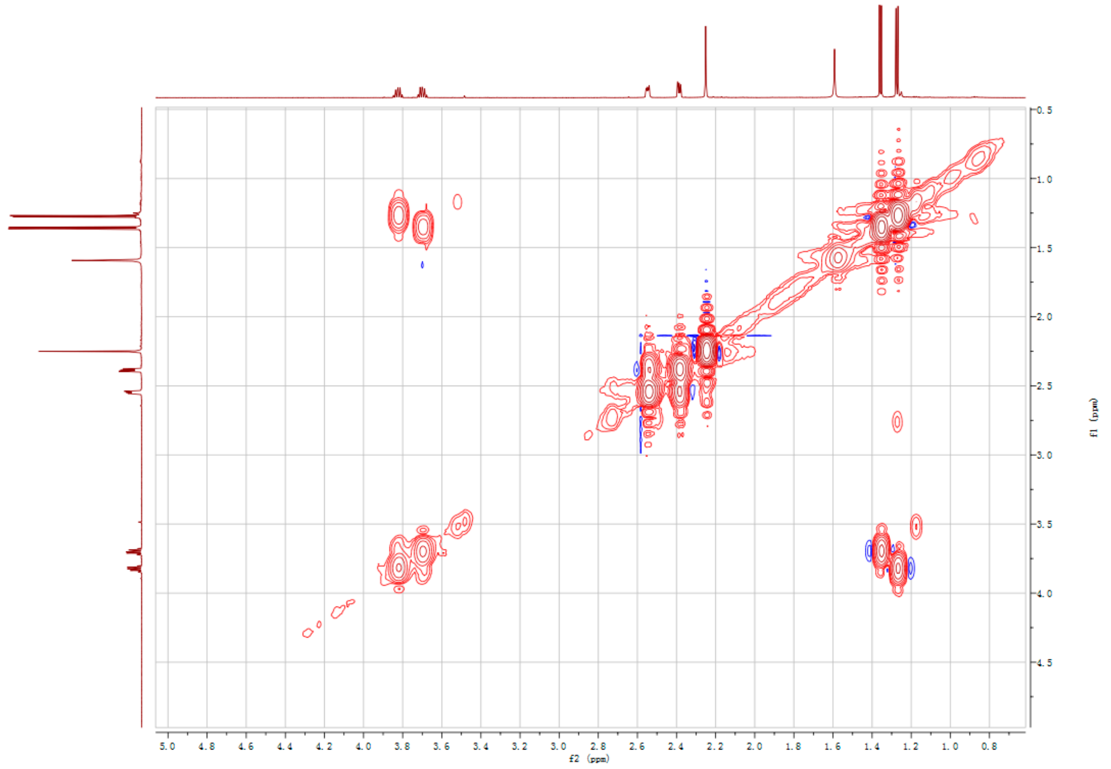

Figure S11. ^{1}H-^{1}H-COSY spectrum of endomeketal B (**2**) in $CDCl_3$.

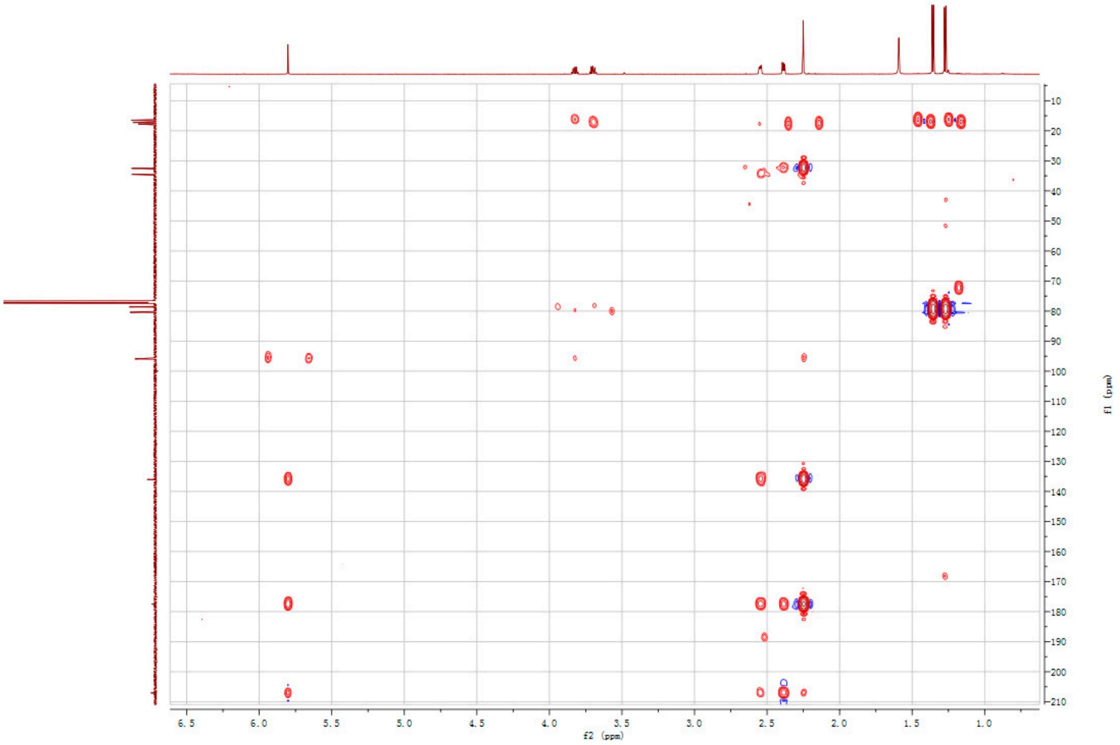

Figure S12. HMBC spectrum of endomeketal B (**2**) in $CDCl_3$.

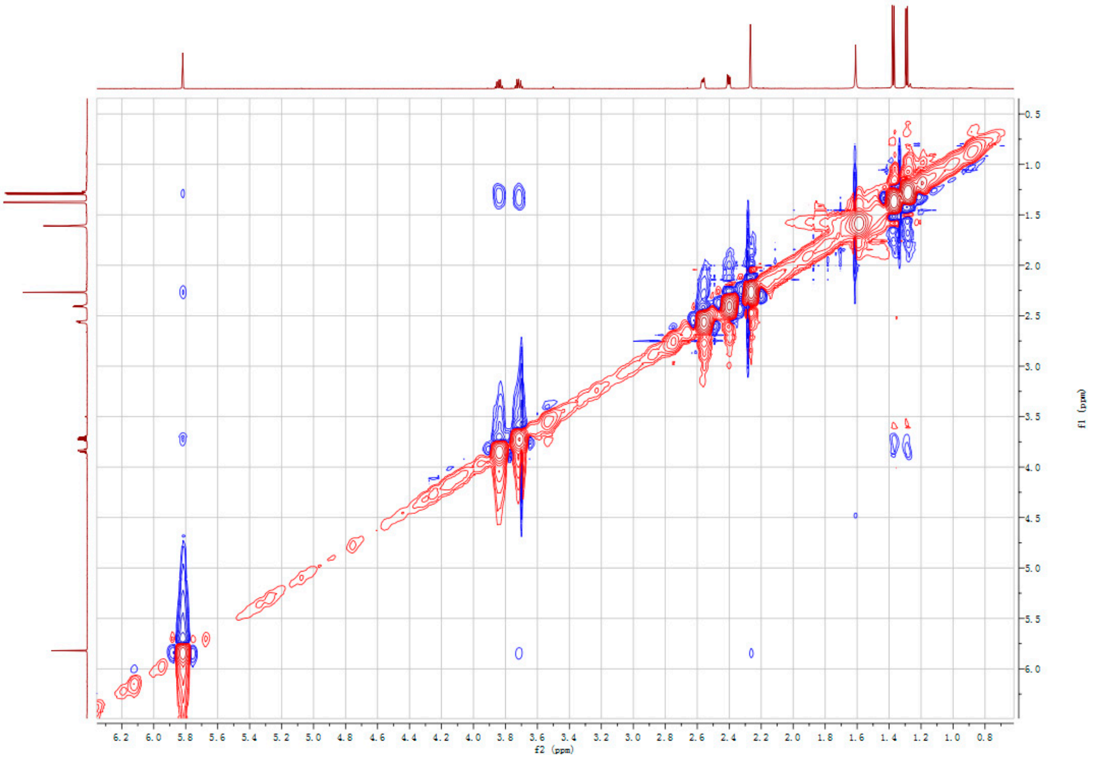

Figure S13. NOESY spectrum of endomeketal B (**2**) in $CDCl_3$.

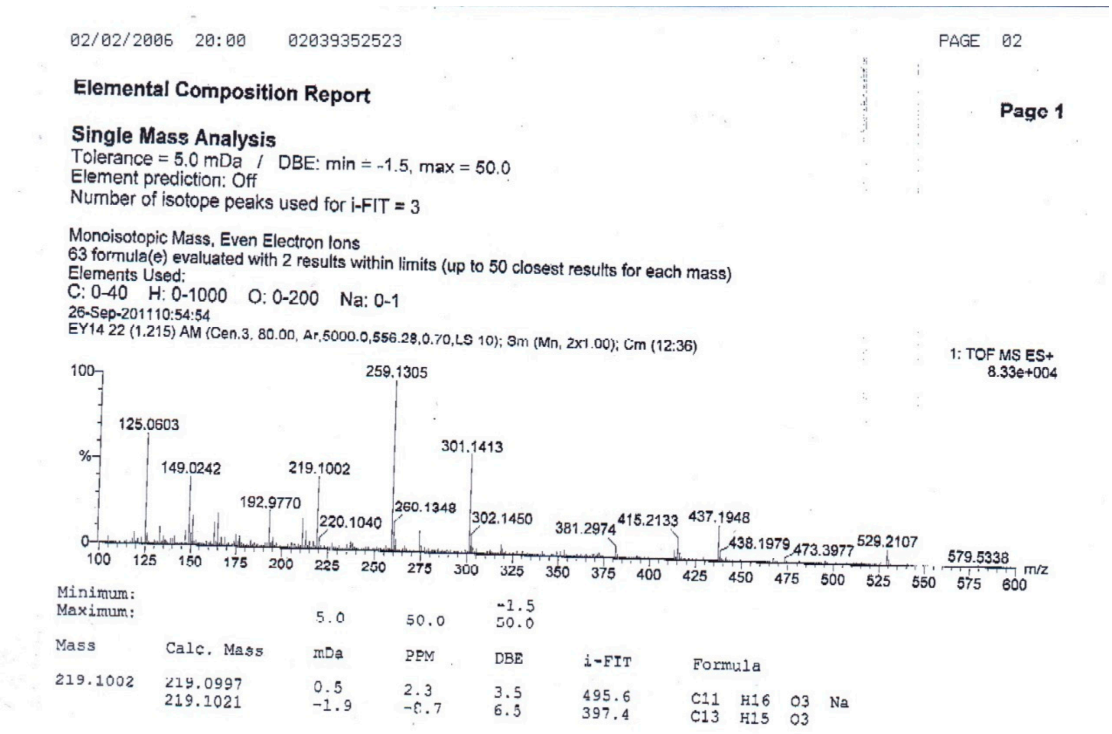

Minimum:				-1.5		
Maximum:		5.0	50.0	50.0		
Mass	Calc. Mass	mDa	PPM	DBE	i-FIT	Formula
219.1002	219.0997	0.5	2.3	3.5	495.6	C11 H16 O3 Na
	219.1021	-1.9	-8.7	6.5	397.4	C13 H15 O3

Figure S14. HRESIMS spectrum of endomeketal B (**2**).

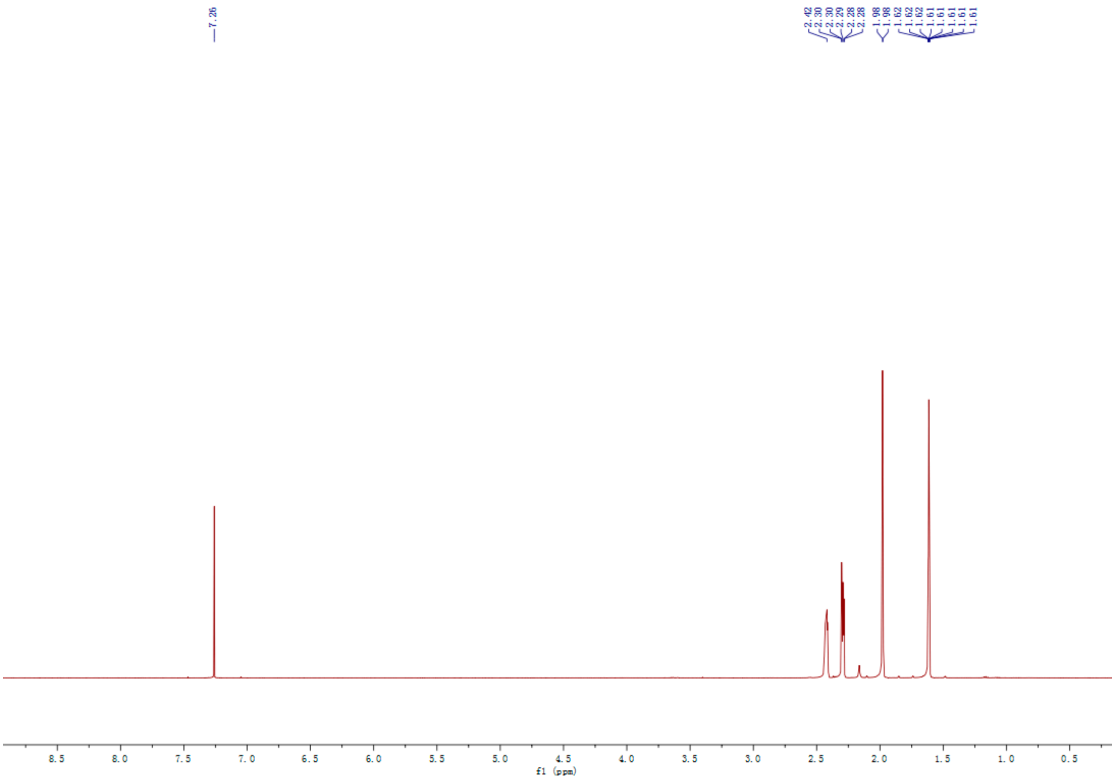

Figure S15. ^{1}H-NMR spectrum of 2,3-dimethylcyclopent-2-en-1-one (**3**) in $CDCl_3$.

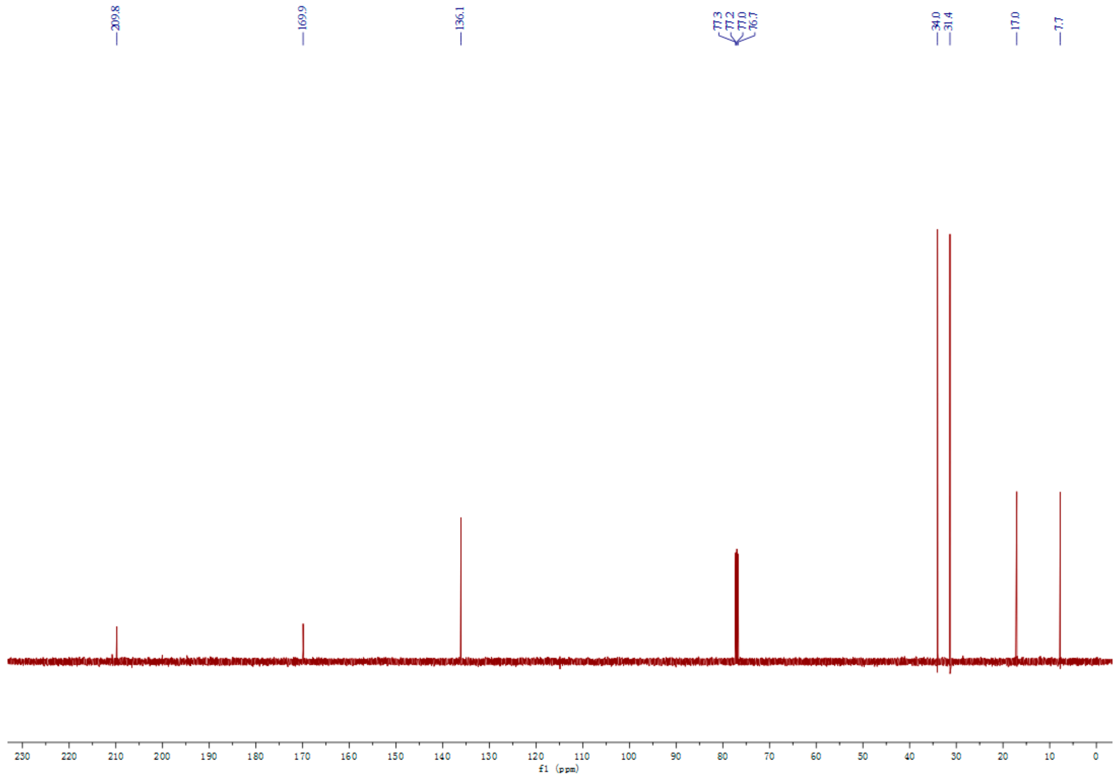

Figure S16. ^{13}C-NMR spectrum of 2,3-dimethylcyclopent-2-en-1-one (**3**) in $CDCl_3$.

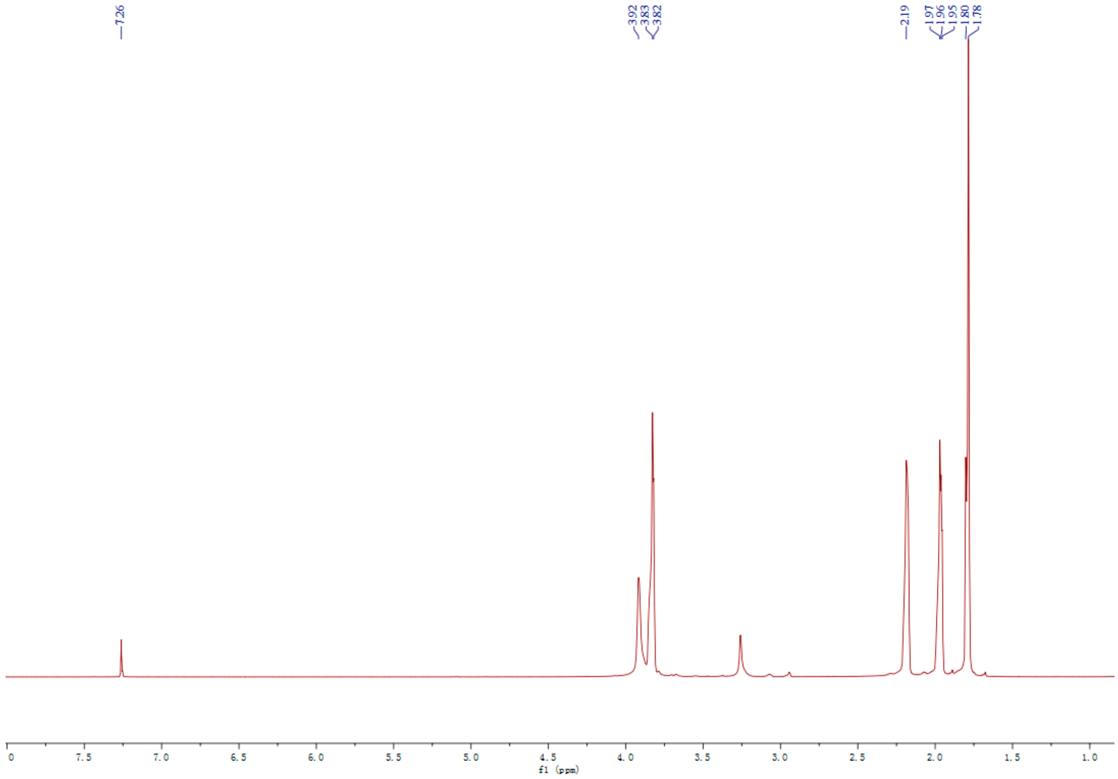

Figure S17. ^{1}H-NMR spectrum of 2-hydroxymethyl-3-methylcyclopent-2-enone (**4**) in $CDCl_3$.

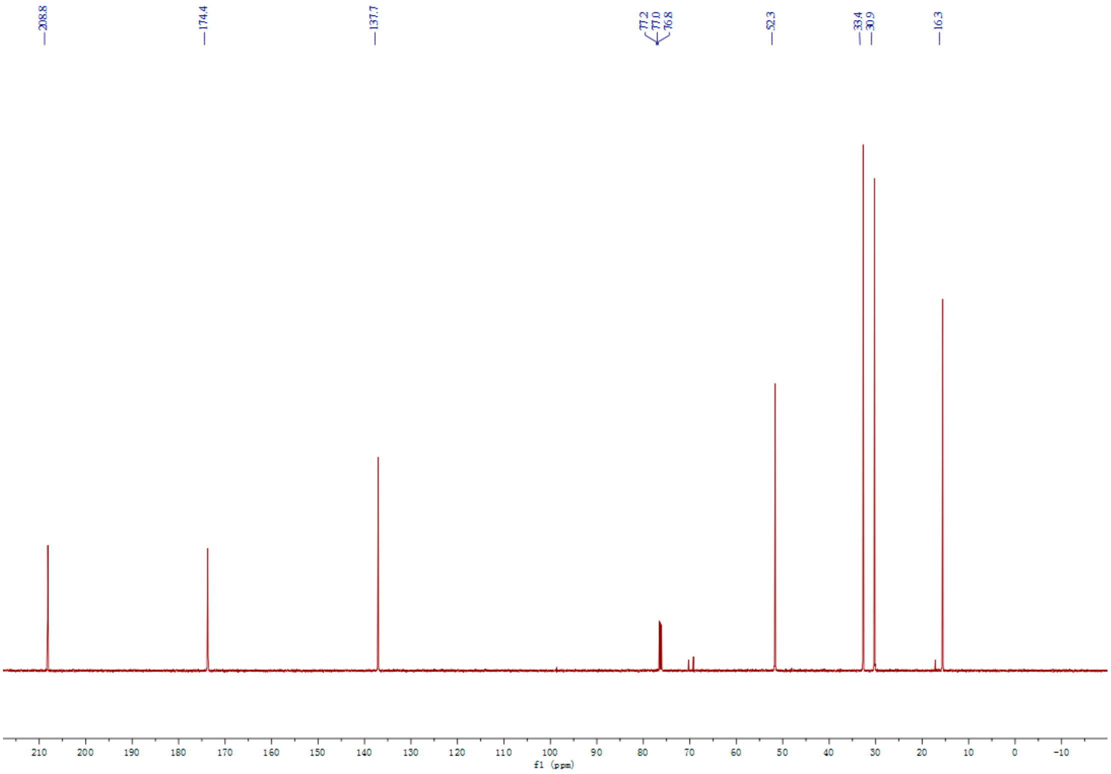

Figure S18. ^{13}C-NMR spectrum of 2-hydroxymethyl-3-methylcyclopent-2-enone (**4**) in $CDCl_3$.
